# Supplementary material for: Tcf4 Is Involved in Subset Specification of Mesodiencephalic Dopaminergic Neurons
Source: Biomedicines. 2021 Mar 20;9(3):317. doi: 10.3390/biomedicines9030317 (PMC8003918; doi:10.3390/biomedicines9030317)
Supplement: Supplementary file 1 [file biomedicines-09-00317-s001.pdf]

Supplemental Figure 1: Expression of TH is not visibly altered in the *Tcf4* mutant at E12.5

Expression of TH (red) appears to be unaffected in the *Tcf4* mutant midbrain compared to WT littermates at E12.5.

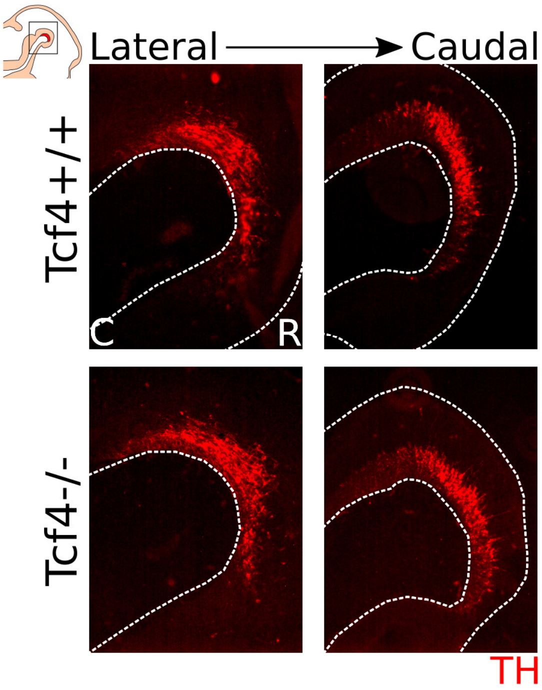

Table S1: qPCR primers sequences of primers used in this study

	Primers		Primers
	FP 5'-ATACTTGTCGGATTTAGGAGGCT-3'		FP 5'-TCGGAGCCAATGTGCAGTCACA-3'
	RP 5'-GGGCCTATCTTCCAAATGAACA-3'		RP 5'-TTGCGTCCTGCCAGCCTCCAT-3'
	FP 5'-TAGCGCGATACATCCAGCAGGT-3'		FP 5'-CTTCCAGAGGAATCGCTACCCT-3'
	RP 5'-GGTATTCGTA		RP 5'-CTGCGAAGC

	GTCCTCGGC ACT-3'		CACCTTTGCA CAG-3'
	FP 5'-GGTGGAAGT CACACTGTC GTAC-3'		FP 5'-AGTCTTCCGC CAATGTCCA G-3'
	RP 5'-GTAACCTCTG GAAGCCGTA GTC-3'		RP 5'-AGCAGCATT CCCTCTGGTT C-3'
	FP 5'-GAGCAAGAA GGCTTGACC CATC-3'		FP 5'-TGACCCTAG CCGGACATA CA-3'
	RP 5'-CCAAACACA ACCTGGAGA CCATC-3'		RP 5'-GGTGTCTCTC CCAAAGGTG G-3'
	FP 5'-CGGAACTGA TGCGCTGCA AACG-3'		FP 5'-AAAGGAATC TGAACCCGA AAG-3'
	RP 5'-GGCAAAACC CAGGTTGAC CAAC-3'		RP 5'-TCACATCTGT CCCATGTGAT -3'
	FP 5'-CTCGGCTTTA ACTGGAGTG C-3'		FP 5'-CAGGCATGG ACCCGATAG GT-3'
	RP 5'-CGAGTCTCGT GTGTTGTCGT -3'		RP 5'-GAAGGAGTA TGCCGGTTTC CC-3'
	FP 5'-TGCTGCTTCG TGTTCCTAGT -3'		FP 5'-CGGTAACAT CGCAGTGTG GGAT-3'
	RP 5'-GTGAGATAT GACCTCGCTT GC-3'		RP 5'-CCTCACAGT GTTGTCCAA ACCAC-3'
